# Real-Time Respiratory Tumor Motion Prediction Based on a Temporal Convolutional Neural Network: Prediction Model Development Study

**DOI:** 10.2196/27235

**Published:** 2021-08-27

**Authors:** Panchun Chang, Jun Dang, Jianrong Dai, Wenzheng Sun

**Affiliations:** 1 Department of Oncology The First Affiliated Hospital of Chongqing Medical University Chongqing China; 2 School of Physics and Electronics Shandong Normal University Jinan China; 3 Department of Radiation Oncology National Cancer Center/National Clinical Research Center for Cancer/Cancer Hospital Chinese Academy of Medical Sciences and Peking Union Medical College Beijing China; 4 Department of Radiation Oncology, School of Medicine The Second Affiliated Hospital Zhejiang University Hangzhou China

**Keywords:** radiation therapy, temporal convolutional neural network, respiratory signal prediction, neural network, deep learning model, dynamic tracking

## Abstract

**Background:**

The dynamic tracking of tumors with radiation beams in radiation therapy requires the prediction of real-time target locations prior to beam delivery, as treatment involving radiation beams and gating tracking results in time latency.

**Objective:**

In this study, a deep learning model that was based on a temporal convolutional neural network was developed to predict internal target locations by using multiple external markers.

**Methods:**

Respiratory signals from 69 treatment fractions of 21 patients with cancer who were treated with the CyberKnife Synchrony device (Accuray Incorporated) were used to train and test the model. The reported model’s performance was evaluated by comparing the model to a long short-term memory model in terms of the root mean square errors (RMSEs) of real and predicted respiratory signals. The effect of the number of external markers was also investigated.

**Results:**

The average RMSEs of predicted (ahead time=400 ms) respiratory motion in the superior-inferior, anterior-posterior, and left-right directions and in 3D space were 0.49 mm, 0.28 mm, 0.25 mm, and 0.67 mm, respectively.

**Conclusions:**

The experiment results demonstrated that the temporal convolutional neural network–based respiratory prediction model could predict respiratory signals with submillimeter accuracy.

## Introduction

The aim of radiation therapy is not only to deliver lethal doses of radiation to target tumors but also to minimize the dose of unnecessary radiation delivered to the surrounding healthy tissues and structures [1-5]. Modern technical advances, such as intensity-modulated radiation therapy, have improved the accuracy of dose delivery. However, some targets, such as lung cancer and liver cancer tumors, may move substantially during the treatment delivery process due to respiratory motion [6-10]. Investigators have reported that lung and liver tumors can move up to 3 cm during a conventional radiation therapy treatment session [11,12]. The motion of targets may substantially decrease the accuracy and efficiency of intensity-modulated radiation therapy or other advanced technologies.

Many methods have been investigated to reduce the effect of respiratory motion, which mainly include the following:

- Adding a margin around the target tumor: a 10- to 15-mm margin is always used as the radiation treatment field to avoid missing a tumor, which may result in unnecessary radiation exposure to heathy tissues and structures [13].
- Breath hold: patients need to hold their breath during the treatment to temporarily stop respiration, but this is not applicable for some patients, such as older patients and juvenile patients [14].
- Beam tracking: radiation beams track a moving tumor dynamically to ensure that the tumor target is constantly within the treatment field [15].

All beam tracking methods must compensate for the latency of various sources, such as latencies from beam adjustment and image capture times [5,16]. Hence, we must estimate the position of targets in advance to compensate for latency effects.

Recently, deep learning approaches based on long short-term memory (LSTM) have been successfully used to solve time series prediction problems in several fields. For example, Ma et al [17] used an LSTM model to capture traffic dynamics data for predicting short-term traffic speed. Bao et al [18] implemented an LSTM model to predict the one-step-ahead price (closing) of 6 stock indices for various financial markets. Lin et al [19] used an LSTM model to predict respiratory signals. Moreover, some recent studies have demonstrated that certain temporal convolutional neural network (TCN) architectures could achieve state-of-the-art accuracy in time series prediction problems [20-23]. However, to our knowledge, there are no studies on using a TCN model to predict respiratory tumor motion. Hence, in this study, we developed a TCN-based respiratory prediction model by using external markers and compared the prediction performance of the TCN to that of an LSTM model. We also investigated the effect that the number of external markers had on prediction performance.

## Methods

### Data Acquisition

The tumor motion data (69 treatment fractions of 21 patients) used in this study were obtained from an open data set, which was recorded by the CyberKnife Synchrony (Accuray Incorporated) tracking system with a recorded sampling rate of 25 Hz [24]. To analyze the external movements of patients, charge-coupled device cameras were used to monitor the luminous diodes located on a patient's abdomen and chest. To analyze internal fiducial positions, orthogonal diagnostic x-ray systems were used to observe implanted markers periodically.

### Prediction Process

**Figure 1:**
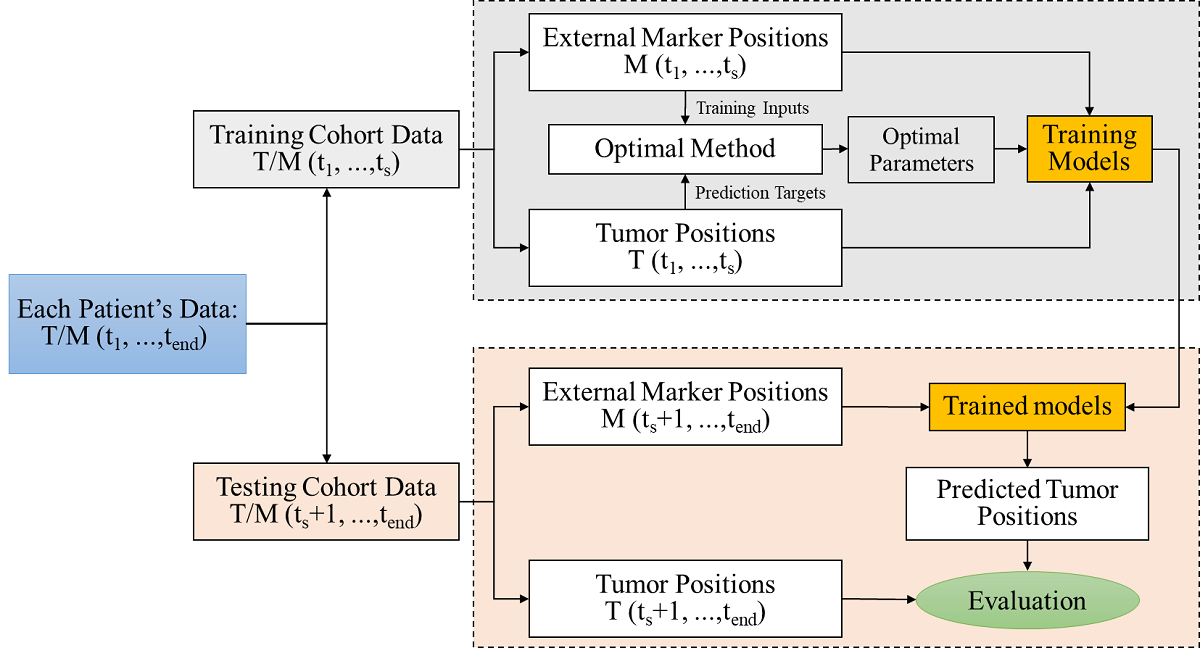
Flowchart of the prediction algorithm.

The general scheme for the prediction process of 2 models is outlined in Figure 1, and the arrangement of the respiratory signals that were used for network training and validation is shown in Table 1. Each recorded position (internal tumor and external marker positions) was stratified into 2 cohorts based on time t_s_. The positions prior to time t_s_ (the training signals) were used to train the TCN and LSTM models. The positions after t_s_ (the testing signals) were used to evaluate the developed model.

**Table 1:** The arrangement of respiratory signals used for network training and validation.

Position type			Data for training		Data for validation
**Inputs of the network**					
	Position of marker 1	M^a^1_SI_^b^, _AP_^c^, _LR_^d^ (1, 2,…, t_s_)		M1_SI, AP, LR_ (t_s_+1, 2,…, t_s_+t_end_)	
	Position of marker 2	M2_SI, AP, LR_ (1, 2,…, t_s_)		M2_SI, AP, LR_ (t_s_+1, 2,…, t_s_+t_end_)	
	Position of marker 3	M3_SI, AP, LR_ (1, 2,…, t_s_)		M3_SI, AP, LR_ (t_s_+1, 2,…, t_s_+t_end_)	
**Targets of the network**					
	Position of a tumor	T^e^_SI, AP, LR_ (1, 2,…, t_s_)		T_SI, AP, LR_ (t_s_+1, 2,…, t_s_+t_end_)	

^a^M: external marker position.

^b^SI: superior-inferior.

^c^AP: anterior-posterior.

^d^LR: left-right.

^e^T: tumor position.

For the training process, the training input data and prediction target data were first used to tune the hyperparameters, which was done by using a cross-validation model. Afterward, they were used to train the model. The external markers’ positions during the first input period of the training process (ie, the time between t=1 and t=t_delay_) were used as the training input data for predicting the tumor positions (target positions) at a specific time frame (t=t_delay_+t_ahead_). This training process was repeated and continued to predict the next tumor position until either the threshold of the cost function or the maximum iteration number, which was set in advance, was reached. Each pair of data points (ie, the input data, M[t+1,…, t+t_delay_], vs the output data, T[t+t_delay_+t_ahead_]) consisted of a training data set. “M” denoted 3 external markers’ positions (M1, M2, and M3), which were based on 3 directions (the superior-inferior, anterior-posterior, and left-right directions). t_ahead_ represented the ahead time we needed for making predictions.

For the evaluation process, the testing signals, which were represented as M(t_s_+1, t_s_+2,…, t_end_) and T(t_s_+1, t_s_+2,…, t_s_+ t_end_), were used to evaluate the developed model. Similar to the process implemented in the training process, the external markers’ positions during the first input period of the evaluation process (ie, the time between t=1 and t=t_delay_) were used to predict a tumor’s position (T’[t_s_+t_delay_+t_ahead_]) at a specific time (t=t_s_+t_delay_+t_ahead_). This process was also repeated to predict the next tumor position continuously. The external signals that were recorded during radiation therapy (ie, the time between t=t_end_−t_delay_−t_ahead_+1 and t=t_end_−t_ahead_) were used to predict the final tumor position (T’[t_end_]). Finally, the predicted signals (T’[t_s_+t_delay_+t_ahead_],…, T’[t_end_]) were compared to the real tumor positions (T[t_s_+t_delay_+t_ahead_],…, T[t_end_]).

### LSTM Model

The recurrent neural network (RNN) is a particular type of neural network that allows for self-cycle connections and transmits parameters across different time stamps. An RNN model can store the information of former time stamps. However, it is difficult for the RNN to memorize long-term memory information due to vanishing and exploding gradients [25-27].

**Figure 2:**
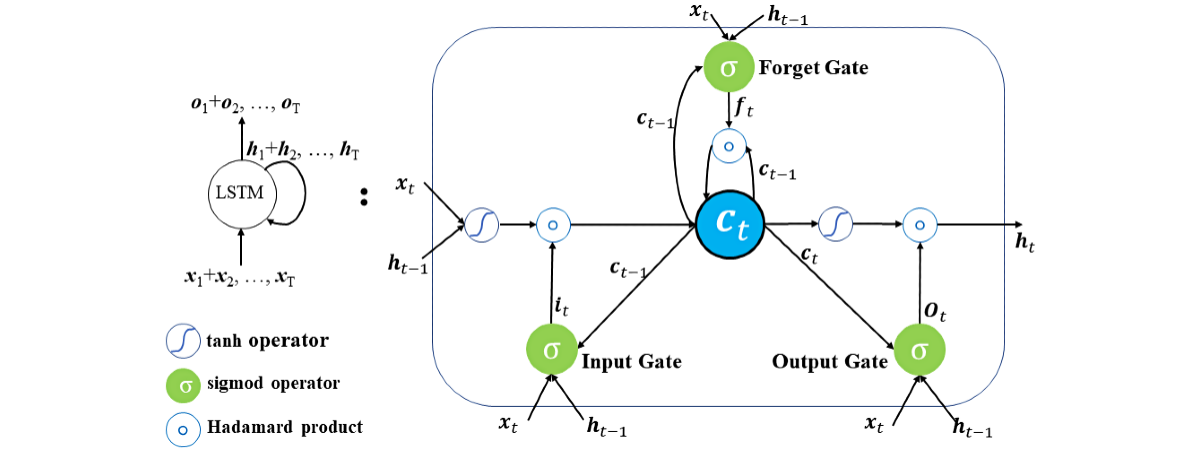
The structure of an LSTM layer. LSTM: long short-term memory.

The LSTM layer is a special RNN layer that overcomes the weakness that the RNN has with memorizing long-term memory information [26,28]. Figure 2 shows an LSTM unit. Unlike the simple RNN unit, the LSTM unit has a memory cell state *c*_t_ at time t. The information that passes through state *c*_t_ is controlled by the following three gates: the input gate (*i_t_*), the forget gate (*f_t_*), and the output gate (*o_t_*). The input gate is used to control input data that flow into state *c*_t_, the hidden state connection (*h_t_*) is used to control the forgetting of state *c*_t_, and the output gate is used to moderate the output data that flow from state *c*_t_. A plurality of LSTM layers can be stacked in a deeper neural network, which can fit the data of the complicated functions that are required to analyze the inputs and the targets.

### TCN Model

**Figure 3:**
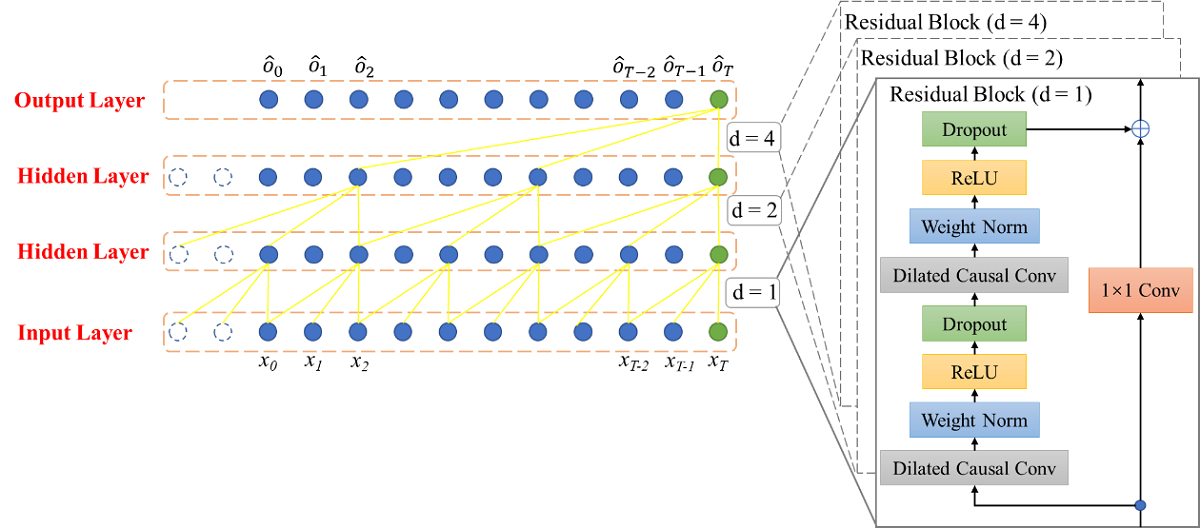
The architecture of the temporal convolutional neural network model. "d" was the dilation factor. Conv: convolution; ReLU: rectified linear unit.

The TCN model was based on a transformation of a 1D fully convolutional network that was used for sequential prediction problems. The TCN model used a multilayer network to learn information over a long time span. Sequence information were transmitted layer by layer across the network until prediction results were obtained. The architecture of the TCN model is illustrated in Figure 3 [23], in which *x_1_*, *x_2_*,…, *x_T_* are the original sequence signals (inputs), and 
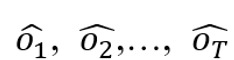
 are the prediction signals (outputs). The obvious characteristics of the TCN model, which were compared to those of the normal 1D fully convolutional network model, were as follows:

- The TCN model used causal convolutions, in which the output at time t was convolved only with elements from previous layers at time t and earlier, to ensure that no leakage occurred from the future into the past.
- The TCN model used dilated convolutions to ensure that each hidden layer had the same size as the input sequence and to increase the receptive field (ie, learning longer lengths of information).

The input of the TCN model was interval sampled. The equation for the dilated convolution was as follows:




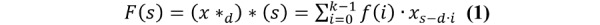




In equation 1, *d* is the dilation factor (sampling rate). A *d* value of 1 in the lowest layer meant that every signal was sampled, whereas a *d* value of 2 in the middle layer meant that every 2 respiratory signals were sampled.

Residual networks [29], which are shown in Figure 3, were imported in this study to accelerate convergence and stabilize training. A residual block that included a branch was used to make a series of transformations (*F*). Afterward, the outputs of the residual block (ie, *F*[X_residual_]) were added to the input (ie, *X*_residual_), as follows:

O_residual_ = Activation(X_residual_ + F[X_residual_])

**(2)**

### Hyperparameter Tuning

With regard to the TCN model, previous TCN studies [20-23] reported (in the *Instruction* section) using the same TCN architecture and only sometimes varying the number of layers (*n*) and the filter size. Hence, we tested these two hyperparameters and used a dilation factor (*d*) of 2*^n^* for layer *n.* Moreover, the number of neurons in the input layer and the learning rate of the TCN model were also investigated in this study. For the LSTM model, the number of LSTM layers, learning rate, number of hidden units per layer, and number of neurons in the input layer were investigated. Furthermore, the Adam algorithm was used as the optimization algorithm for both the TCN model and LSTM model. The Kingma and Ba [30] study demonstrated that the hyperparameters in the Adam model required little tuning. Goodfellow et al [31] also approved of the robustness of the Adam model for their hyperparameter of choice and provided advice on how to tune the learning rate from the default value. Hence, we used the good default settings that were tested by Kingma and Ba [30] as the hyperparameters of the Adam optimizer and tuned the learning rate. The default settings were exponential decay rates of 0.9 and 0.999 and a decay exponent of 10^−8^. In this study, all hyperparameters were tuned synthetically by using a grid search model. It should be noted that we tested the hyperparameters in a 4D hyperparameter space instead of a subspace (ie, while a parameter was investigated, others were fixed) to maintain the accuracy of hyperparameter tuning.

### Model Evaluation

The respiratory signals from 69 treatment fractions of 21 patients with cancer who were treated with the CyberKnife Synchrony (Accuray Incorporated) device were used to evaluate the proposed model. Of the 69 treatment fractions, 5 were used to tune the hyperparameters. The rest of the patients were used to evaluate prediction performance. For each of the 69 treatment fractions, signals that were acquired around the first 3 minutes (4500 data points) were used as the training signals for training the prediction model, and signals from the following 30 seconds were used as the test signals for assessing the effectiveness of the proposed model. The ahead time (t_ahead_) used in this study was 400 ms [1,5].

The root mean square errors (RMSEs) between real and predicted signals of respiratory motion in a 3D space were used for assessment [6,7]. The RMSEs for motion in each direction (*RMSE_SI, LR, AP_*) and motion in a 3D space (*RMSE_3D_*) were calculated by using equations 3 and 4, respectively, as follows:




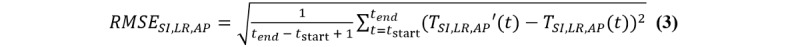







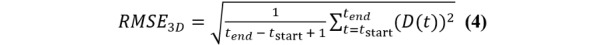







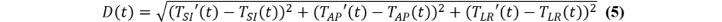




In equation 5, 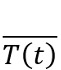 is the average of the true values, and 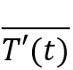 is the average of predicted values. Time point t in equation 3 ranged from t_start_ (*t_s_*+t*_delay_*+t*_ahead_*) to t_end_. The Wilcoxon signed-rank test was used as the statistical model for evaluating the differences between true values and predicted values.

## Results

Table 2 presents the RMSEs of the three models (ie, the LSTM, TCN, and no prediction models; ahead time=400 ms). Compared to the no prediction model, the RMSEs for motion in a 3D space were reduced by 46% in the LSTM model and 51% in the TCN model. For motion in all directions, the RMSEs of the TCN model were consistently lower than those of the LSTM model. The RMSE for motion in a 3D space decreased from 0.73 mm (LSTM model) to 0.67 mm (TCN model). The *P* value was <.001, indicating that the TCN method could significantly improve the prediction performance of the LSTM method.

**Table 2:** The root mean square errors (RMSEs) of the three prediction models.

Direction	RMSEs (mm) of the LSTM^a^ model	RMSEs (mm) of the TCN^b^ model	RMSEs (mm) of the no prediction model
Anterior-posterior direction	0.29	0.28	0.50
Left-right direction	0.27	0.25	0.45
Superior-inferior direction	0.55	0.49	1.04
3D space	0.73	0.67	1.36

^a^LSTM: long short-term memory.

^b^TCN: temporal convolutional neural network.

**Figure 4:**
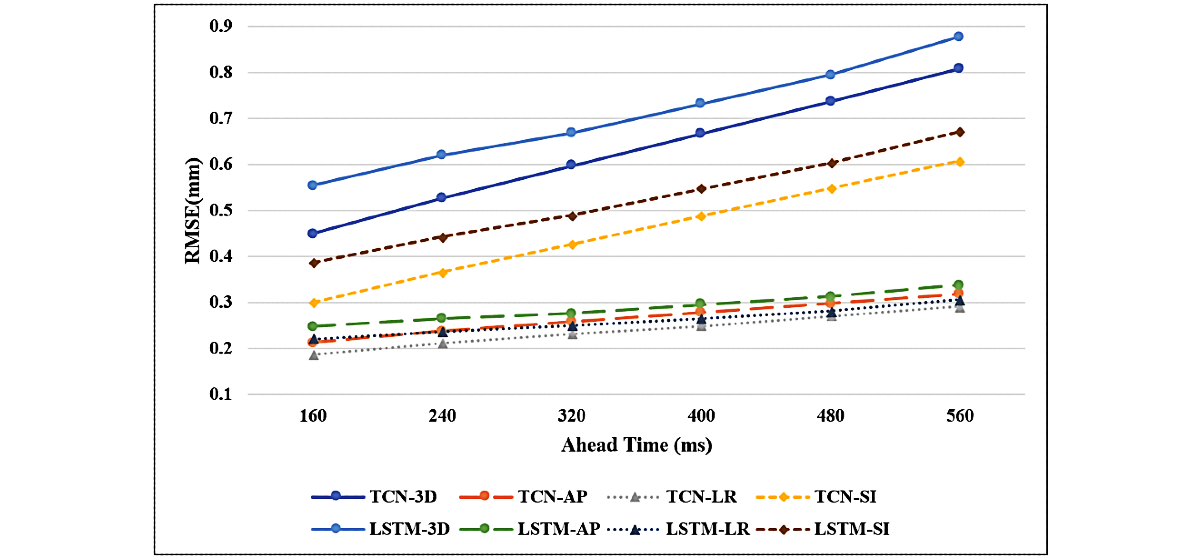
The RMSEs for respiratory motion in all directions. These were determined by using the LSTM and TCN models and different ahead times for each treatment fraction. AP: anterior-posterior; LR: left-right; LSTM: long short-term memory; RMSE: root mean square error; SI: superior-inferior; TCN: temporal convolutional neural network.

Figure 4 shows the RMSEs for motion in all directions with different ahead times. Obviously, the prediction performance of the TCN model was positive compared to that of the LSTM model for all ahead times. Further, the prediction performance of both models worsened as ahead times increased.

**Figure 5:**
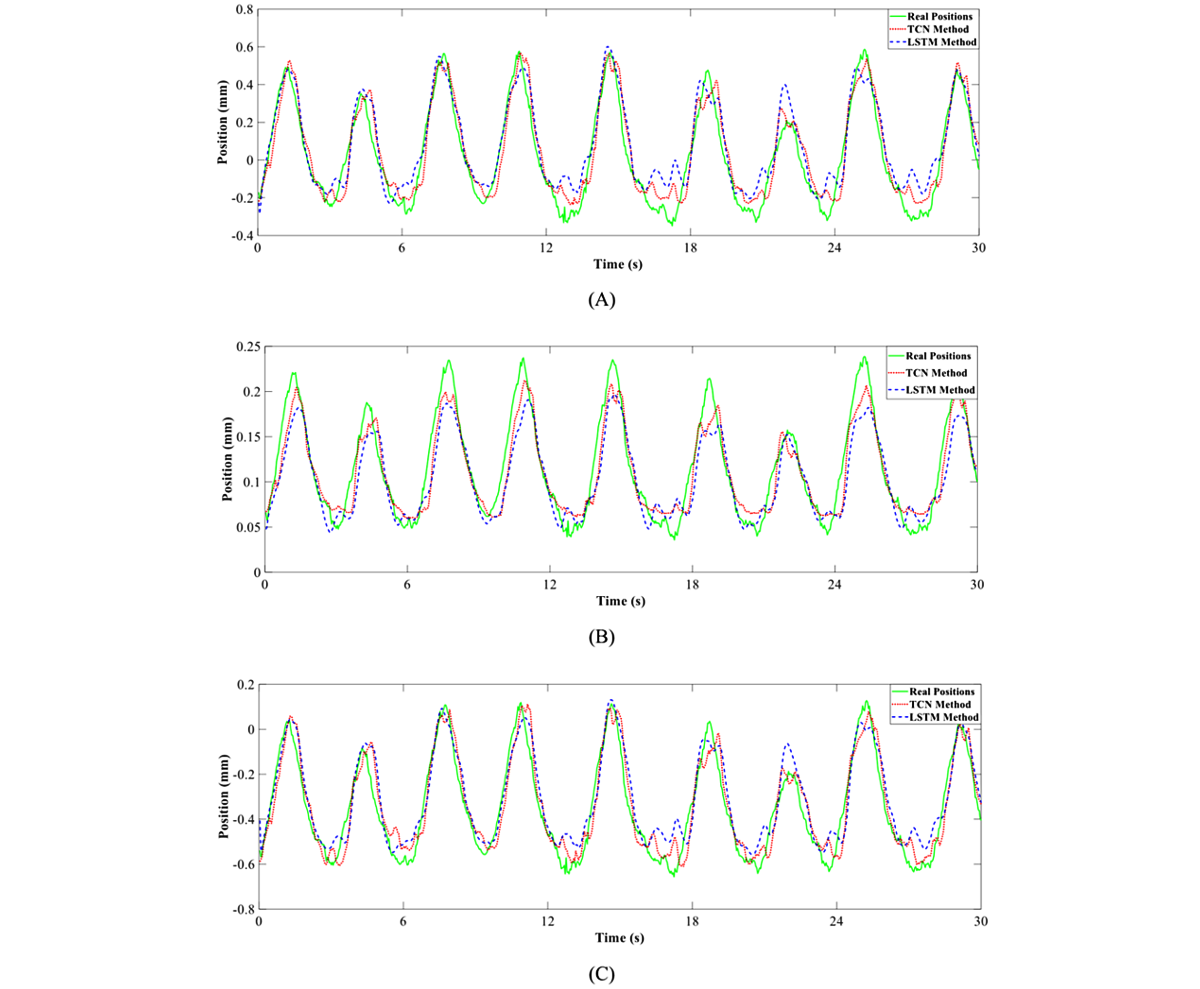
The performance comparison between the TCN and LSTM methods for predicting motion in the (A) superior-inferior direction, (B) left-right direction, and (C) anterior-posterior direction. LSTM: long short-term memory; TCN: temporal convolutional neural network.

Figure 5 illustrates the performance comparison between the TCN and LSTM methods for predicting motion in the superior-inferior direction, anterior-posterior direction, and left-right direction. Obviously, the TCN method was more accurate and robust than the LSTM method.

We investigated the hyperparameters in the 4D hyperparameter space (625 experiments) for both the TCN and LSTM models by using the grid search method among 5 treatment fractions, which were selected randomly. The options and results of hyperparameter tuning are depicted in Table 3.

**Table 3:** The options and results of hyperparameter tuning.

Models and hyperparameters			Hyperparameter options		Hyperparameter selected
**Temporal convolutional neural network model**					
	Number of layers	4, 5, 6, 7, and 8		5	
	Filter size	1, 3, 5, 7, and 9		9	
	Number of neurons in the input layer	5, 10, 15, 20, and 25		15	
	Learning rate	0.0001, 0.001, 0.005, 0.01, and 0.1		0.001	
**LSTM^a^ model**					
	Number of LSTM layers	1, 2, 3, 4, and 5		2	
	Learning rate	0.0001, 0.001, 0.005, 0.01, and 0.1		0.01	
	Number of hidden units per layer	10, 50, 100, 150, 200, and 250		200	
	Number of neurons in the input layer	5, 10, 15, 20, and 25		20	

^a^LSTM: long short-term memory.

**Figure 6:**
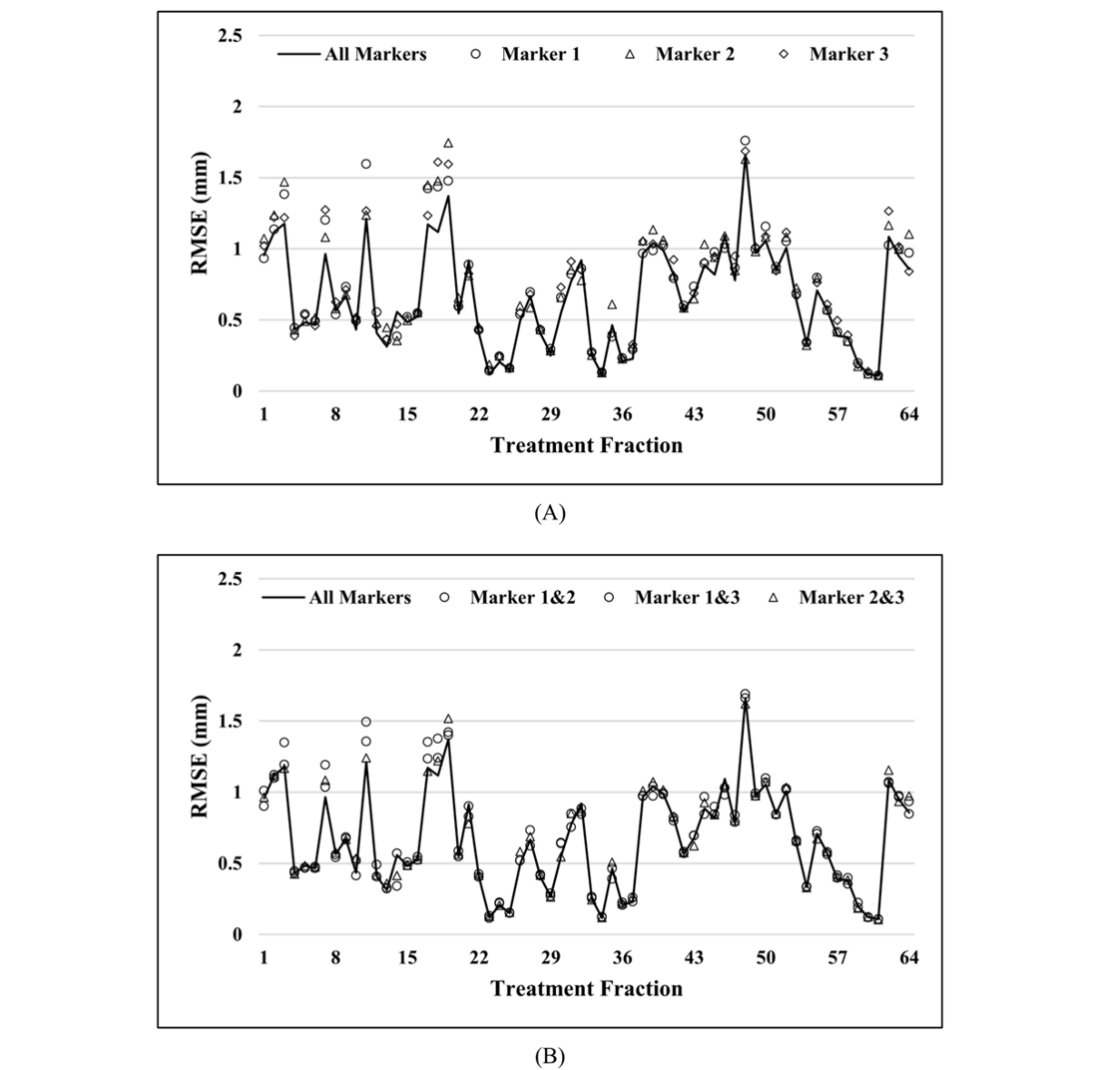
A comparison of RMSEs for respiratory motion in a 3D space among each treatment fraction. A: Results of the TCN model using 1 external marker compared to those of the TCN model using all 3 external markers. B: Results of the TCN model using 2 external markers compared to those of the TCN model using all 3 external markers. RMSE: root mean square error; TCN: temporal convolutional neural network.

Table 4 presents the RMSEs of the TCN model for each external marker. Figure 6 shows the RMSEs for respiratory motion in a 3D space among each treatment fraction. The TCN model using 1 or 2 external markers was compared to the TCN model using all 3 external markers. The TCN model had the best performance in terms of predicting motion in all directions when all three external markers were used simultaneously. The average RMSEs for motion in a 3D space when using 1 marker and 2 markers were 0.72 mm and 0.68 mm, respectively. This decreased to 0.67 mm when using all three makers.

**Figure 7:**
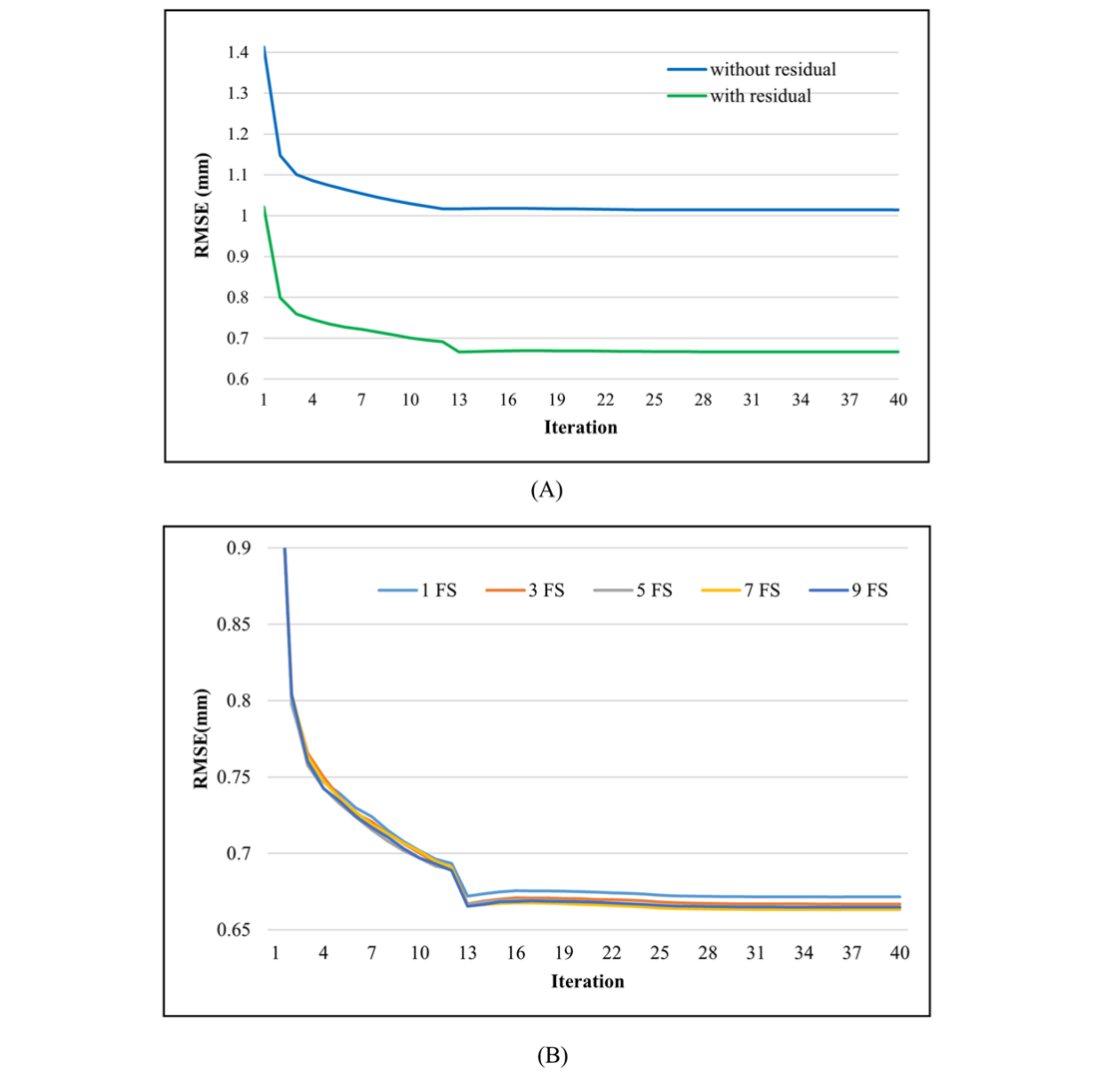
The effects of different components in the temporal convolutional neural network layer. A: Residual blocks. B: FS. FS: filter size; RMSE: root mean square error.

As illustrated in Figure 7, the ablative analysis of the TCN was also conducted. We focused on two components in this study—the filter size and the residual blocks. We found that the effect of the filter size was small when the filter size was larger than 3. The *P* values between 5 filter size pairs—filter sizes 1 and 3, 3 and 5, 5 and 7, and 7 and 9—were <.001, .11, .20, and .83, respectively. This indicated that prediction performance improved significantly before the filter size rose to 3. Further, we found that the residual blocks contributed significantly to prediction performance, as the *P* value was <.001.

**Table 4:** The root mean square errors (RMSEs) of the temporal convolutional neural network model for each external marker (EM).

Direction	RMSEs for all EMs	RMSEs for EMs 1 and 2	RMSEs for EMs 1 and 3	RMSEs for EMs 2 and 3	RMSEs for EM 1	RMSEs for EM 2	RMSEs for EM 3
Anterior-posterior direction	0.28	0.28	0.28	0.28	0.29	0.29	0.29
Left-right direction	0.25	0.26	0.26	0.25	0.27	0.26	0.26
Superior-inferior direction	0.49	0.51	0.50	0.50	0.52	0.53	0.53
3D space	0.67	0.69	0.68	0.68	0.71	0.72	0.72

## Discussion

### Principal Findings

A TCN model for predicting respiratory motion by using external markers’ prior signals was developed and tested in this study. The experiment demonstrated that the TCN model’s performance in predicting future respiratory signals with a 400-ms ahead time was better than that of the LSTM model.

As is well known, hyperparameter settings have a large influence on the prediction performance of machine learning models. This also holds true for our TCN and LSTM models. We tuned 4 major hyperparameters for both of the TCN and LSTM models. Among these hyperparameters, the number of neurons in the input layer and the learning rate were tested for both models. Having a large number of neurons in the input layer allows for the inclusion of more features in models. Obviously, useful features may increase prediction accuracy. However, redundancy features may also be brought in along with the useful features. Hence, if this hyperparameter is too large, prediction performance may degenerate. The best number of neurons in the input layer for the TCN and LSTM models in this study was 15 and 20, respectively. The learning rate was an important hyperparameter in the model optimization process. If the learning rate is too large, the model may oscillate around the global minimum value instead of achieving convergence. On the other hand, if this value is too small, the training time and the risk of overfitting increase. Learning rates of 0.001 and 0.01 were selected as the optimal hyperparameters of the TCN and LSTM models, respectively. In addition to the two abovementioned hyperparameters, the number of layers and filter sizes were also investigated for the TCN model, whereas the number of LSTM layers and number of hidden units per layer were tested for the LSTM model. With regard to the TCN model, the size of the effective window (receptive field) increased as the number of layers and filter size increased. Hence, these two hyperparameters should guarantee that the receptive field of TCN model covers enough context for respiratory signal prediction. The optimal values for these two hyperparameters in our experiments were 5 and 9, respectively. With regard to the LSTM model, on one hand, a deeper LSTM model (a large number of LSTM layers) may be representative of a more complicated relationship among respiratory signals and improve prediction performance. On the other hand, a deeper LSTM model also has an increased risk of overfitting and increased convergence speed. In this study, the prediction performance results of the LSTM model were comparable when the number of LSTM layers was over 2. Hence, we selected 2 as the optimal number of LSTM layers. Further, the number of hidden units per layer determined the width of each LSTM layer. We also found that having a large number of hidden units per layer was helpful for establishing a more complicated prediction model, but at the same time, this increased the risk of overfitting and convergence speed.

The effect that different numbers of external markers had on prediction performance was also investigated in this study. The TCN model had the best prediction performance when it used all three markers’ positions. As shown in Figure 6, the TCN model’s prediction performance when using 3 markers was more robust than when using 1 marker or 2 markers. For most treatment fractions, the RMSEs of the TCN model using 3 markers was slightly smaller than those obtained by using 1 marker or 2 markers. However, for some treatment fractions, such as treatment fractions 7 and 11, the RMSEs of predictions based on 1 or 2 external markers were quite larger than those of predictions based on 3 external markers. This was probably because having more external markers for different skin surface positions resulted in the inclusion of more useful features. Such useful features may alleviate the overfitting and underfitting problems.

Finally, we studied the influence of the different components (the filter size and residual blocks) in the TCN model. The size of the effective window (receptive field) increased with filter size. Hence, the model’s prediction performance initially became better as the filter size increased. However, the model’s prediction performance only slightly improved as the filter size increased continually. This may be because the receptive field that resulted from using a filter size of 3 provided enough context for the respiratory signal prediction task. On the other hand, we observed that the residual block architecture enhanced the model’s prediction performance immensely. We believe that this was because the residual blocks effectively allowed the TCN model to be modified based on identity mapping instead of a full transformation, which was crucial for the deep neural network architecture.

### Conclusion

A deep learning approach based on the TCN architecture was developed to predict internal tumor positions with a 400-ms ahead time based on the external markers’ positions in this study. The results demonstrated that this model could predict tumor positions accurately. Further, the prediction performance of the TCN model using multiple external markers was more robust and positive than that of the TCN model using 1 or 2 external markers.

## Abbreviations

LSTM: long short-term memory
RMSE: root mean square error
RNN: recurrent neural network
TCN: temporal convolutional neural network
